# Far-Reaching Dispersal of *Borrelia burgdorferi* Sensu Lato-Infected Blacklegged Ticks by Migratory Songbirds in Canada

**DOI:** 10.3390/healthcare6030089

**Published:** 2018-07-25

**Authors:** John D. Scott, Kerry L. Clark, Janet E. Foley, Bradley C. Bierman, Lance A. Durden

**Affiliations:** 1 International Lyme and Associated Diseases Society, Bethesda, MD 20827, USA; 2 Epidemiology & Environmental Health, University of North Florida, Jacksonville, FL 32224, USA; kclark@unf.edu (K.L.C.); bradleybierman@gmail.com (B.C.B.); 3 Vector-borne Disease Epidemiology and Department of Veterinary Medicine, University of California, Davis, CA 95616, USA; jefoley@ucdavis.edu; 4 Department of Biology, Georgia Southern University, Statesboro, GA 30458, USA; ldurden@georgiasouthern.edu

**Keywords:** Lyme disease, *Borrelia burgdorferi* sensu lato, blacklegged ticks, *Ixodes scapularis*, songbirds, bird migration, northern Canada

## Abstract

Lyme disease has been documented in northern areas of Canada, but the source of the etiological bacterium, *Borrelia burgdorferi* sensu lato (Bbsl) has been in doubt. We collected 87 ticks from 44 songbirds during 2017, and 24 (39%) of 62 nymphs of the blacklegged tick, *Ixodes scapularis*, were positive for Bbsl. We provide the first report of Bbsl-infected, songbird-transported *I. scapularis* in Cape Breton, Nova Scotia; Newfoundland and Labrador; north-central Manitoba, and Alberta. Notably, we report the northernmost account of Bbsl-infected ticks parasitizing a bird in Canada. DNA extraction, PCR amplification, and DNA sequencing reveal that these Bbsl amplicons belong to *Borrelia burgdorferi* sensu stricto (Bbss), which is pathogenic to humans. Based on our findings, health-care providers should be aware that migratory songbirds widely disperse *B. burgdorferi*-infected *I. scapularis* in Canada’s North, and local residents do not have to visit an endemic area to contract Lyme disease.

## 1. Introduction

Lyme disease is caused by members of *Borrelia burgdorferi* sensu lato (Bbsl) complex, and this spirochetal bacterium is typically transmitted to vertebrates by certain ixodid (hard-bodied) ticks (Acari: Ixodidae) [1]. East of the Rocky Mountains, the primary vector of Bbsl is the blacklegged tick, *Ixodes scapularis* Say, which is an ectoparasite of more than 125 species of North American vertebrates (avian, mammalian, reptilian) [2]. This tick species commonly parasitizes passerine birds (Passeriformes) and, recently, in eastern Canada, has been found parasitizing an American Kestrel, *Falco sparverius* Linnaeus, a small-size raptor [3]. Historically, Scott et al. provide the first report of a Bbsl-infected tick on a bird in Canada [4], and motile spirochetes were isolated from an *I. scapularis* nymph collected from a passerine migrant on Bon Portage Island, Nova Scotia. Blacklegged tick larvae and nymphs are known to feed on 81 bird species. In central and eastern Canada, Bbsl infection prevalence for songbird-transported *I. scapularis* nymphs ranges from 31–35% [5,6]. Blacklegged ticks are known to harbour at least 10 tick-borne pathogens, and engorging females may cause tick paralysis.

Worldwide, the Bbsl complex consists of at least 23 genospecies. In Canada, six genospecies have been detected in ixodid ticks, and these genospecies comprise: *B. americana* [7,8], *B. burgdorferi* sensu stricto (Bbss) [9,10], *B. kurtenbachii* [11], *B. lanei* (formerly *Borrelia* genospecies 2) [12,13], *B. garinii* [9,14], and *B. bissettiae* (formerly *B. bissettii*) [15,16,17].

Migratory songbirds are very mobile, and have exceptionally flight capability to transport bird-feeding ticks long distances during bidirectional migrations. DeLuca et al. reported a Blackpoll Warbler, *Setophaga striata* (Forster) flying 923 km/d during a 3-day, non-stop, overwater flight during autumn migration [18]. In addition, *Amblyomma* species ticks (*A. americanum*, *A. dissimile*, *A. humerale*, *A. longirostre*, *A. maculatum*, *A. rotundatum*) have been transported into Canada from as far south as Brazil [4,19,20,21]. Previously, bird-feeding *I. scapularis* immature ticks have been reported as far north and as far west as the municipality of Slave Lake, Alberta [4,19,21]. Furthermore, Lyme disease vector ticks have been reported on spring passerine migrants as far north as Watson Lake, Yukon [21]. Here, we present the first records of Bbsl-positive *I. scapularis* in several northern regions of Canada; these findings demonstrate the wide geographic dispersal of Lyme disease vector ticks by migratory songbirds in northern latitudes of North America.

## 2. Materials and Methods

### 2.1. Tick Collection

**Figure 1:**
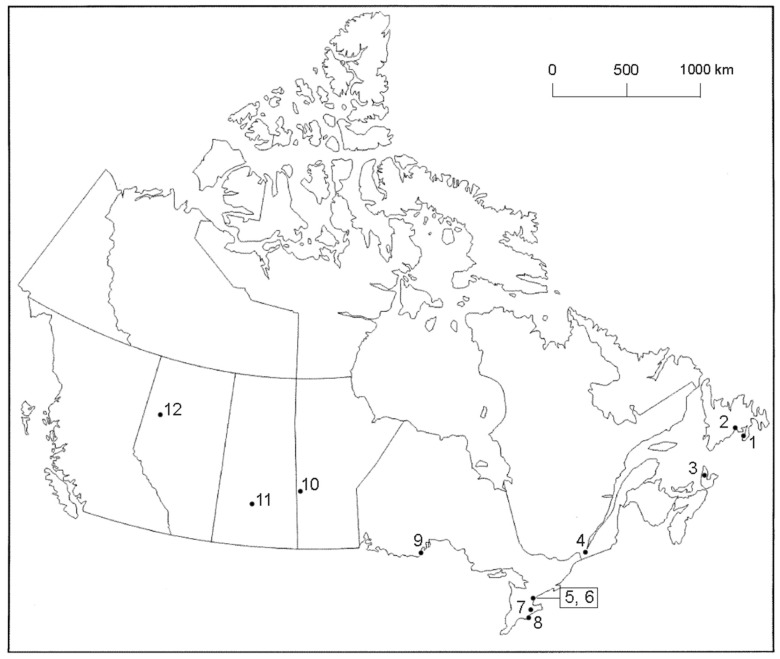
Collection sites across Canada where ixodid ticks were collected from songbirds, 2017. (1) Grand Bank, Newfoundland and Labrador, 47.10° N, 55.75° W; (2) St. Albans, Newfoundland and Labrador, 47.86° N, 55.84° W; (3) Middle River, Cape Breton Island, Nova Scotia, 46.19° N, 60.92° W; (4) Ste-Anne-de-Bellevue, Quebec, 45.40° N, 73.95° W; (5) Toronto, Ontario (Tommy Thompson Park Bird Research Station), 43.63° N, 79.33° W; (6) Toronto, Ontario (Fatal Light Awareness Program), 43.63° N, 79.42° W; (7) Ruthven Park, Ontario (Cayuga), 42.98° N, 79.87° W; (8) Long Point, Ontario (Port Rowan), 42.52° N, 80.17° W; (9) McKellar Island, Ontario (Thunder Bay), 48.19° N, 89.13° W; (10) Swan River, Manitoba, 52.11° N, 101.27° W; (11) Saskatoon, Saskatchewan, 52.13° N, 106.67° W; and (12) Peace River, Alberta, 56.23° N, 117.29° W. The locations in parentheses represent mailing addresses.

Ticks were collected from songbirds by bird banders, wildlife rehabilitators, biologists, citizen scientists, Fatal Light Awareness Program staff, and the public who captured and rescued songbirds at 12 locations from Alberta to Newfoundland and Labrador (Figure 1). Fine-pointed, stainless steel forceps were used for tick removal. These ticks were placed in vented vials and, then, placed in ziplock plastic bags with slightly moistened paper towel, and sent directly to the lab (J.D.S.) for identification. Tick species, developmental life stage, and extent of engorgement were determined [22,23,24]. Live, fully engorged ticks were held to molt in a special housing unit with 95% humidity. In late summer, when daylength shortened, we used a full spectrum light bulb (FC LifeLite, LED 12W) on a timer for an extended photoperiod of 16:8 h. (L:D).

### 2.2. Molecular Tick Identification

In order to confirm the identification of a representative tick (17-5A8), 8 legs from this tick were barcoded at the Centre of Biodiversity Genomics (CBG), University of Guelph with accession number BIO-17-124. The sample ID is: BIOUG35422-H11. The DNA extract is being held at −80 °C at the same location. The collection data and barcode sequence is stored on the Barcode of Life Datasystems (BOLD; www.boldsystems.org), and can be accessed in the BOLD dataset at: dx.doi.org./10.5883/DS-IGAK. The nucleotide sequence has been deposited in GenBank with the accession number: MG521417.

### 2.3. Spirochete Detection

The ticks were divided into 2 lots, and sent to 2 separate molecular laboratories for Bbsl testing. In Lab F (J.E.F.), Bbsl DNA was extracted from ticks, and tested for Bbsl via real time PCR (RT-PCR) using methods previously described by Barbour et al. [25]. We selected the PCR primer that was specifically designed to identify *B. burgdorferi* sensu lato. Sterile water was used for negative controls, and positive controls consisted of DNA from cultured Bbss. Samples were considered positive if the cycle threshold (CT) was <40, and there was a characteristic amplification curve. However, for DNA sequencing, RT-PCR positive samples were re-tested to amplify a portion of the conserved 41-kDa chromosomal flagellin (*flaB*) gene using primers described by Clark et al. [26]. The two primers for the *flaB* gene were: (1) Fla OutF: AAR-GAA-TTG-GCA-GTT-CAA-TC and (2) Fla OutR: GCA-TTT-TCW-ATT-TTA-GCA-AGT-GAT-G.

In Lab C (K.L.C.), all extracts were also screened for the presence of Bbsl *flaB* DNA by using nested PCR as described previously [13]. For verification of the presence of Bbsl DNA, *flaB* positive samples were sequenced in both directions with forward and reverse primers used in the nested PCRs. Representative sequences from positive samples were deposited in the GenBank database.

## 3. Results

### 3.1. Tick Collection

**Figure 2:**
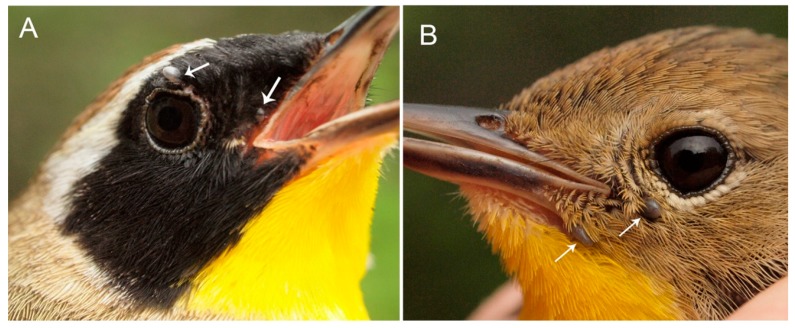
Common Yellowthroat: (**A**) male parasitized by 3 *I. scapularis* nymphs, 17-5A42, of which 1 is not visible; all nymphs were positive for *B. burgdorferi* sensu stricto. (**B**) female parasitized by 2 *I. scapularis* nymphs, 17-5A39. Photo credits: Simon Duval.

During 2017 (8 April to 3 October), we collected 87 ticks from 44 songbirds consisting of 20 bird species (Table 1), from 12 sites across Canada from Alberta to Newfoundland and Labrador (Figure 1). Of these ixodid ticks, 84 were tested for Bbsl. The bird with the most frequent parasitism by host-seeking ticks was the Common Yellowthroat (*n* = 10) (Figure 2), and it was followed next by the Swainson’s Thrush (*n* = 5). With exception of one location (Site 11), we obtained Bbsl-positive ticks from passerines from all sites (Table 2). Although we recorded low tick numbers on passerines in Saskatchewan, all ixodid ectoparasites collected in this province were the bird-rabbit tick, *Haemaphysalis leporispalustris*.

### 3.2. Molecular Tick Identification

**Figure 3:**
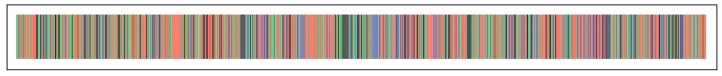
Illustrative barcode of the *Ixodes scapularis* specimen TJDD017-17 generated in the Spider package [27].

Using a taxonomic key [24], the preliminary identification of the nymph (17-5A8) was *I. scapularis*. Likewise, the *Ixodes* female, following its nymph-adult molt, was *I. scapularis* [23]. The specimen (TJSD017-17) was successfully sequenced for the DNA barcode region of COI, with a sequence length of 658 base pairs. Comparison with the DNA barcode library using the BOLD ID Engine resulted in 99.67–99.85% pairwise nucleotide matches to over 100 *I. scapularis* specimens housed in BOLD, further confirming its identification (Figure 3). The barcode sequence is available at GenBank with the following accession: MGS521417.

### 3.3. Spirochete Detection

Overall, 24 (28%) of 87 ticks tested were positive for Bbsl (Table 1). The Bbsl infection prevalence for *I. scapularis* nymphs was 24/62 (39%). All of the *H. leporispalustris* and *I. affinis* ticks tested were negative for Bbsl. Using the flagellin (*flaB*) gene, DNA sequencing revealed that all Lyme borreliae belong to Bbss. The northernmost location where Bbsl-positive *I. scapularis* immature ticks were collected from migratory songbirds was Peace River, Alberta. This locality is on the flight path for many neotropical and southern temperate songbirds that breed and rear their young in the northern boreal forest.

Blacklegged tick nymphs were collected from a Swainson’s Thrush, *Catharus ustulatus*, on 26 May 2017 at Swan River, Manitoba. The 3 nymphs molted to adults (1 male, 2 females) in 51, 55, and 59 days, respectively. Both females were positive for Bbsl. When these 2 borrelial amplicons were sequenced, they were characterized as Bbss.

Several *B. burgdorferi*-positive *I. scapularis* ticks were collected at Ste-Anne-de Bellevue, Québec. In one particular collection, all 3 *I. scapularis* nymphs (17-5A42) from a Common Yellowthroat, *Geothlypis trichas*, collected on 20 May 2017, were positive for Bbsl (Figure 2). Bird species that were parasitized by *B. burgdorferi* s.l.-infected *I. scapularis* ticks collected in Québec include the Northern Waterthrush, *Parkesia noveboracensis*, Northern House Wren, *Troglodytes aedon*, and Common Yellowthroat.

Three fully engorged *I. scapularis* nymphs were collected from a Swainson’s Thrush on 27 May 2017 at Peace River, Alberta. They were held to molt to adults (2 females, 1 male) in 47, 52 and 55 days. One female (17-5A80A) and one male (17-5A80B) were positive for Bbsl.

### 3.4. Spirochete Amplicon Sequences

We sequenced PCR-amplified *flaB* gene fragments from 21 of 24 Bbsl-positive samples, and compared the sequences to those obtained via BLAST (Basic Local Alignment Search Tool) by searching the GenBank database (Table 2). The sequences were trimmed to the same length (367 nucleotides long) from base position 356 through to the base position 722 of the Bbsl *flaB* gene, and compared to the reference strain B31 (sequence ID X15661 in GenBank). All of the tick-derived Bbsl *flaB* sequences were between 99% and 100% similar to the B31 strain sequence with 0, 1, or 2 nucleotide differences.

## 4. Discussion

This study presents evidence of ground-foraging songbirds transporting Lyme disease vector ticks to northern latitudes in Canada. We have documented Bbsl in *Ixodes* ticks parasitizing migratory songbirds collected from Alberta to Newfoundland and Labrador. Some of these neotropical and southern temperate migrants have wintering ranges in the Caribbean, Central and South America. These long-distance migrants provide insights into how people in northern areas contract Lyme disease. In this study, we detected a Bbsl infection prevalence of 39% in songbird-transported *I. scapularis* nymphs which is slightly higher than previous bird-tick-pathogen studies that ranged from 31–35% [5,6]. Although Lyme disease vector ticks have been reported as far north as the Yukon [21], we report the first Bbsl-positive *Ixodes* ticks parasitizing migratory songbirds in several northern regions of Canada.

### 4.1. Photoperiod to Molt Blacklegged Ticks

When we obtained fully engorged *I. scapularis* larvae and nymphs from fall passerine migrants, we found that they would not molt unless they had more than 14 h of daylength. Even though these replete ticks were held at room temperature (21 °C), they would not molt unless they had extended daylength. In order to have these larval and nymphal ticks molt to the next life stage, we had to use a full spectrum light bulb on a timer with a photoperiod of 16:8 h (L:D). Photoperiod is critically important in determining whether *I. scapularis* can establish at any given latitude [28].

### 4.2. Obstacles Facing Migratory Songbirds

In this study, we obtained bird-feeding ticks in several different ways, especially during northward spring migration. Some migratory songbirds, such as the Willow Flycatcher, *Epidonax traillii*, may fly 8000 km or more between wintering and breeding grounds [29]. Migration requires high energy reserves, and enhances exposure to predators; thus, it is typically a period of high mortality. Migratory songbirds are susceptible to predatory cats, power transmission lines, vehicles, hunting, agricultural pesticides, houses and tall buildings. Blancher [30] calculated that outdoor feral and domestic cats kill more than 196 million birds annually in Canada, making them the most significant bird mortality factor. In our study, we collected ticks from 11 different ground-frequenting songbirds either by rescuing birds from domestic cats or birds obtained after collisions with moving vehicles or with tall buildings with reflective glass. When songbirds collide with lighted skyscrapers and glass facades, they normally fall to the ground, and often predator birds, such as gulls, prey on these helpless, stunned birds. In some urban locales, Fatal Light Awareness Program volunteers come to the rescue and rehabilitate birds and, at the same time, collect host-seeking ticks. Specifically, 2 of 3 engorged, *I. scapularis* nymphs collected on 2 May 2017 from a Northern House Wren, which collided with a multi-story buildings in downtown Toronto, were spirochetemic for Bbsl. Some researchers suggest that 80% of annual bird mortalities occur during migration [31].

### 4.3. Bird Parasitism during Spring Migration

The migratory flight path of many songbirds corresponds with Lyme disease endemic areas in the northern U.S.A., and likewise, with several Lyme disease foci in central and northern Canada. While en route to more northerly breeding grounds, especially the boreal forest, these north-bound passerines make landfall at stopovers to replenish their energy reserves. As these ground-foraging songbirds look for food, they are often parasitized by *I. scapularis* immatures, especially nymphs. Peak questing activity of *I. scapularis* nymphs, in May and early June, corresponds closely with peak northward migration of many neotropical and southern temperate migrants [32]. After these bird-feeding ticks have taken a blood meal, they are subsequently dispersed in tick-supportive habitats across Canada.

### 4.4. Spirochete-Positive Blacklegged Ticks in Central Manitoba

Our findings reveal that Bbsl-positive host-seeking *I. scapularis* immatures are being transported by migratory songbirds to mid-central latitudes of Manitoba. These findings are consistent with other bird-tick-pathogen studies [5,6,21], which documented migratory songbirds transporting Lyme disease-carrying ticks in Manitoba during the annual northward spring migration.

### 4.5. Bird-Feeding Ticks Positive for Lyme Disease Spirochete in Québec

Our results show that passerines are dispersing blacklegged tick larvae and nymphs in Québec, some of which are infected with Bbsl. Since transovarial transmission (female-egg transfer) of Bbsl does not occur in *I. scapularis* ticks [33], the Bbsl-positive nymphs must have acquired infection when larvae fed on Bbsl-infected hosts or directly from its avian host.

### 4.6. Songbird-Transported Ticks Infected with Lyme Disease Bacterium in Alberta

We provide the first report of Bbsl-positive *I. scapularis* ticks parasitizing a bird in Alberta. Previously, tick researchers have documented *I. scapularis* immature ticks in this province [4,19]. When the amplicons underwent DNA sequencing, they were delineated as Bbss. However, there was considerable heterogeneity between two Bbss strains detected in these two Bbsl-positive nymphs. It is likely that the initial spirochetal infections occurred at two different locations when these ticks were in their larval stage. Alternatively, the host bird may have been harbouring two different Bbss strains, and transmitted them directly to the nymphs during their blood meals. The Bbsl-positive *I. scapularis* female has the potential to infect any suitable mammalian host, including humans.

At this northern latitude (56.23° N), the daylength is less than 14 h in late August. Consequently, immature stages of *I. scapularis* will not molt to the next life stage in late summer. A photoperiod of >14 h is required for *I. scapularis* larvae and nymphs to molt [28]. Therefore, *I. scapularis* cannot become established in this northern location or further north. However, songbird-transported *I. scapularis* immatures could molt in early summer after spring migration and, after molting, could bite humans and other suitable hosts.

In retrospect, Bbsl was detected in *H. leporispalustris*, collected from a snowshoe hare, *Lepus americanus*, at Grande Prairie, Alberta [34]. Clearly, Bbsl is present in the environment in Alberta, and migratory songbirds facilitate long-distance dispersal of Bbsl-infected ticks, especially during the spring and fall migration. In addition, researchers have reported immature stages of Lyme disease vector ticks (i.e., *I. pacificus*, *I. scapularis*, *I. spinipalpis*) parasitizing songbirds in Alberta [4,19,21], and each of these tick species exhibits vector competency [35]. These epidemiological findings provide sound evidence of how people can contract Lyme disease and associated tick-borne diseases in this province.

### 4.7. Ticks Positive for Lyme Disease Spirochete in Cape Breton, Nova Scotia

Three engorged *I. scapularis* nymphs were collected from a Common Yellowthroat on 28 May 2017 at Middle River, Cape Breton, Nova Scotia. These nymphs molted to adults (2 males, 1 female) in 43, 48, and 55 days. The Lyme borreliae were characterized as Bbss. Notably, we provide the first record of Bbsl-positive ticks on a bird captured in Cape Breton. It is noteworthy that the first Bbsl-infected tick on a bird in Canada was collected in southern Nova Scotia [4].

### 4.8. Ticks Infected with Lyme Disease Spirochete in Newfoundland and Labrador

Three engorged *I. scapularis* nymphs were collected from a Common Yellowthroat on 28 May 2017 at Grand Bank, Newfoundland and Labrador. These nymphs molted to adults (2 males, 1 female) in 37, 44, and 49 days. Two of the 3 adults tested positive for Bbsl and, when sequenced, were characterized as Bbss. This novel bird parasitism constitutes the first record of a Bbsl-positive tick on a bird in Newfoundland and Labrador. In addition, 2 engorged *I. scapularis* nymphs were collected from a Gray-cheeked Thrush, *Catharus minimus*, on 29 May 2017 at St. Albany, Newfoundland and Labrador. Both nymphs molted to females in 38 and 45 days. One *I. scapularis* female was positive for Bbsl; it was characterized as Bbss.

### 4.9. Songbirds Establish Tick Populations

Tick-laden songbirds have the capacity to initiate new foci of ticks hundreds of kilometres from their original source. Any heavily infested songbird, which is parasitized by Bbsl-infected *I. scapularis*, has the makings to establish a new population of *I. scapularis* ticks in a suitable habitat [36,37,38]. Oftentimes, migratory songbirds will make stop-overs at Lyme disease endemic areas, and become parasitized by Lyme disease vector ticks. In the present study, a Common Yellowthroat (tick no. 17-5A42), which was parasitized by the 3 Bbsl-positive *I. scapularis* nymphs, and has the means to start a Lyme disease endemic area. As well, neotropical songbirds transport ticks from as far south as Brazil, and import them into Canada during northward spring migration [39,40]. After these ticks molt, they have the potential to infect suitable hosts in northern latitudes.

Some passerine migrants are parasitized by *I. scapularis* immatures that are co-infected with more than 1 tick-borne pathogen. Hersh et al. detected 3 different pathogens (*Anaplasma phagocytophilum*, *Babesia microti*, *Borrelia burgdorferi*) in an *I. scapularis* nymph collected from a Veery, *Catharus fuscescens* [41]. This triple co-infection shows that birds have the potential to be parasitized by ticks harbouring a wide range of tick-borne pathogens, such as *Babesia duncani* [42], and subsequently, these tick-associated pathogens can be transmit to other vertebrates. Such bird parasitisms reveal how numerous tick-borne pathogens can be introduced into a breeding colony of *I. scapularis* ticks. Similarly, people can be co-infected with multiple zoonotic pathogens during a tick bite.

### 4.10. Viability of B. burgdorferi Sensu Lato in Bird-Feeding Ticks

The viability of Bbsl in this study has been questioned because ticks were not cultured. Culturing and sub-culturing isolates is a very time-consuming and labour-intensive task, and this procedure is commonly bypassed. Contamination of cultures by unwanted microorganisms often occurs, and is a laboratory bugbear. Regardless of whether culturing is successful or not, nucleic acid testing, namely PCR, is ultimately employed to detect and identify Bbsl.

In this study, many of the fully engorged *I. scapularis* ticks completed the nymph-adult molt before they were preserved. During the molt, the midgut of *I. scapularis* larva and nymphs remains intact during development of the next life stage; the rest of the tick exoskeleton, including the foregut and hindgut, is shed at the end of the molt [43]. Lyme disease spirochetes reside in the midgut, and remain viable throughout the molt.

When *I. scapularis* larvae and nymphs molt, Bbsl remains viable and infective during the typical 5–8 week molt, and the resulting nymphs and females are able to infect subsequent hosts. Any *I. scapularis* ticks, which tested positive for Bbsl in the present study, would have been harbouring motile spirochetes prior to the 94% ethyl alcohol preservation. Notably, *I. scapularis* has transstadial transmission of Bbsl and, when this tick species becomes infected, retains Lyme disease spirochetes for the rest of its life. 

In previous bird-tick-pathogen studies, Lyme disease spirochetes were isolated from host-seeking ticks collected from songbirds during spring migration [4,5,19,21]. The only difference between these earlier bird-tick-pathogen studies, and the present study, is the timeframe. Despite our not culturing ticks, 39% of the *I. scapularis* nymphs were positive for Bbsl. The bird-feeding ticks in the present study would, no doubt, have been infected with motile Lyme disease spirochetes just before preservation in 94% ethyl alcohol.

In mammalian hosts, non-viable Lyme disease spirochetes are promptly shed by the body. When Straubinger et al. inoculated the skin of beagles with heat-killed Bbsl, the borrelial fragments were no longer present after 3 wk [44]. Notably, the molt period in *I. scapularis* ticks is significantly longer than the time for clearance of Bbsl from mammalian hosts. Blacklegged ticks retain viable Bbsl bacteria in the midgut, whereas mammalian hosts promptly shed them.

Based on culturing of Bbsl from *I. scapularis* nymphs in previous studies, and the ability of Bbsl to remain in ticks during the current study, we have substantive evidence that Bbsl bacteria in *I. scapularis* ticks are infective. Even though we preserved ticks for PCR testing, we are assured that if they were kept alive, they would have been able to transmit infective, Lyme disease-causing spirochetes to people.

### 4.11. Implications of Human Lyme Disease

Because Lyme disease is a zoonosis, it is pathologically important to provide an interconnecting link between vector ticks and humans. These ectoparasites have super-sensitive sensory organs that detect urine, carbon dioxide, and phenols given off by potential hosts [45]. Ticks can transmit innumerable pathogens to people during engorgement, and these pathogenic microorganisms typically cause multisystem infections, including Lyme disease.

When Bbsl-infected *I. scapularis* bite, they commonly transmit Lyme disease spirochetes in 24–48 h [46]; however, Cook [47] reported transmission in less than 16 h, particularly if the tick salivary glands are infected. Whenever *I. scapularis* ticks harbour other tick-borne pathogens, such as *Anaplasma phagocytophilum* (the causative agent of human anaplasmosis), they can often transmit these pathogens in less than 24 h [48]. Additionally, Powassan virus can be transmitted in less than 15 min [49]; thus, there is no grace period between tick attachment and transmission. Because *Babesia* sporozoites reside in tick salivary glands, they can be transmitted immediately when the tick starts to take a blood meal [50].

After transmission, spirochetes disseminate throughout the body, and lodge in tissues and organs. Lyme disease patients may have an erythema migrans rash (i.e., bull’s-eye, homogenous, erythema multiforme, atypical); however, 40% or less have erythematous rashes [51,52,53,54]. Only 14% of Lyme disease patients recall a tick bite [55]. As spirochetes advance in the body, patients typically experience a wide array of symptoms, including fatigue, flu-like symptoms, muscle aches and pain, radicular pain, arthritis, peripheral neuropathy, cognitive impairment, increased impulsivity, sensory hypersensitivity (to sound, touch, smell, taste and/or light), and intense emotional lability [56]. Spirochetes evade and slip by host defenses, lodge intracellularly, and form more resilient forms, such as biofilms [57,58]. These stealth pathogens also attach to, invade and kill B and T lymphocytes [59]. When Bbsl bacteria are killed off, the byproducts (biotoxins) induce inflammatory cytokines (i.e., interleukin 1, interleukin 6, TNF-alpha) [60,61]. These biotoxins typically cause fever, muscle ache and pain, headaches, cognitive impairment, and sometimes, skin discoloration [61,62]. For instance, acrodermatitis chronica atrophicans shows up as skin discoloration on body extremities [62]. At an elevated level, biotoxins can cause tick paralysis [63,64]. In addition, borrelial biotoxins will induce mitochondrial dysfunction, oxidative stress, hormonal abnormalities, depressive tendencies, and neuropsychiatric manifestations [61,65]. Patients will often experience a Jarisch-Heixheimer reaction when treatment is initiated [66,67,68].

If left untreated or inadequately treated, Bbsl will sequester and persist in deep-seated tissues including brain [69,70,71], collagenous tissue (i.e., ligament, tendon) [72,73], bone [74], eye [75], muscle [76], glial and neuronal cells [77,78], synovium [79], and fibroblasts/scar tissue [80]. In addition, live Bbsl spirochetes have been cultured from human blood after the patient was bitten by a blacklegged tick [81,82]. Since Bbsl is pleomorphic, these diverse forms (i.e., spirochete, blebs, granules, spherocytes) facilitate intracellular *Borrelia* sequestration in these tissues [83]. Collectively, these aggregates combine to form slime-coated, polysaccharide matrices, called biofilms, and exacerbate persistence of infection [58]. Bbsl has persister cells and sleeper cells, which are known to survive antimicrobials, and subsequently, recrudescence of infection may occur [68,84,85,86,87]. Since Lyme disease spirochetes are in human testicles, seminal and vaginal secretions, this spirochetosis has the potential to be sexually transmitted [87,88].

Early treatment is paramount; delayed treatment may be arduous and challenging [89,90], and result in fatal outcomes [69,91]. Lyme disease can have traumatic social and psychological effects on patients, partners, and family that include extreme temper tantrums, increased irritability, oppositional behaviors, aggressiveness, violence, suicide, homicidality, and homicide [92,93].

In conclusion, we report the northernmost locations in North America, where bird-feeding, Bbsl-positive ticks have been collected. Passerine migrants widely disperse Bbsl-infected *I. scapularis* from Alberta to Newfoundland and Labrador, and people do not have to visit an endemic area to contract Lyme disease and associated tick-borne diseases. Our flagship findings reveal that 39% of the songbird-transported *I. scapularis* nymphs are positive for Bbsl, and these spirochetes belong primarily to *B. burgdorferi* sensu stricto, which is pathogenic to humans. Based on our data, we provide substantive evidence to show how people in northern latitudes of Canada may contract Lyme disease via the bite of a Bbsl-infected, songbird-transported ticks. The medical profession should be cognizant that *Ixodes* ticks infected with Lyme disease spirochetes are a major public health risk across Canada.

## Figures and Tables

**Table 1:** Detection of *Borrelia burgdorferi* sensu lato in ixodid ticks collected from songbirds in Canada, 2017.

Bird Species	No. of Birds	*Hlp*			*Iaf*		*Imu*			*Isc*		No. Ticks	No. Ticks
		L	N		N		L	F		L	N	Collected	Pos/Tested (%)
Eastern white-crowned Sparrow, *Zonotrichia leucophrys* (Forster)	1	-	-		0/1		-	-		-	-	1	0/1 (0)
Song Sparrow, *Melospiza melodia* (Wilson)	2	-	-		-		-	-		0/1	1/1	2	1/2 (50)
Brown Thrasher, *Toxostoma rufum* (L.)	1	-	-		-		-	-		-	1/1	1	1/1 (100)
Northern House Wren, *Troglodytes aedon* (Vieillot)	4	-	-		0/1			0/3		-	3/5	9	3/9 (33)
Hermit Thrush, *Catharus guttatus* (Pallas)	1	-	-		-		-	-		-	0/1	1	0/1 (0)
Swamp Sparrow, *Melospiza georgiana* (Latham)	1	-	-		-		-	-		-	0/1	1	0/1 (0)
Ovenbird, *Seiurus aurocapillus* (L.)	2	-	-		-		-	-		-	0/2	2	0/2 (0)
White-throated Sparrow, *Zonotrichia albicollis* (Gmelin)	3	-	-		-		-	0/1		-	1/4	5	1/5 (20)
Northern Waterthrush, *Parkesia noveboracensis* (Gmelin)	2	-	-		-		-	-		-	1/3	3	1/3 (33)
Common Yellowthroat, *Geothlypis trichas* (L.)	10	-	-		0/2		-	-		-	8/17	19	8/19 (42)
Magnolia Warbler, *Setophaga magnolia* (Wilson)	1	-	-		-		-	-		-	0/2	2	0/2 (0)
Veery, *Catharus fuscescens* (Stephens)	2	-	-		0/1		-	-		0/1	0/3	5	0/5 (0)
Gray-cheeked Thrush, *Catharus minimus* (Lafresnaye)	2	-	-		-		-	-		-	1/2	2	1/2 (50)
Swainson’s Thrush, *Catharus ustulatus* (Nuttall)	5	5 ^†^	1 ^†^		-		-	-		0/2	6/9	17	6/11 (55)
Indigo Bunting, *Passerina cyanea* (L.)	1	-	-		-		-	-		-	1/4	4	1/4 (25)
Mourning Warbler, *Geothlypis philadelphia* (Wilson)	1	-	-		-		-	-		-	0/2	2	0/2 (0)
Dark-eyed Junco, *Junco hyemalis* (L.)	1	-	-		-		-	-		-	1/2	2	1/2 (50)
Lincoln’s Sparrow, *Melospiza lincolnii* (Audubon)	2	-	-		-		-	-		-	0/3	3	0/3 (0)
Chipping Sparrow, *Spizella passerina* (Bechstein)	1	0/4	0/2		-		-	-		-	-	6	0/6 (0)
Gray Catbird, *Dumetella carolinensis* (L.)	1	-	-		-		2 ^‡^	-		-	-	2	0/1 (0)
Total: 20 species	44	14	3		5		3	4		4	24/62 (39)	89	24/87 (28)

L, larva(e); N, nymph(s); F, female(s); *Hlp*, *Haemaphysalis leporispalustris*; *Iaf*, *Ixodes affinis*; *Imu*, *Ixodes muris*; *Isc*, *Ixodes scapularis*; ^†^, none tested; ^‡^, one tested, one not tested.

**Table 2:** Associations of *Borrelia burgdorferi* sensu stricto-infected *Ixodes scapularis* nymphs and songbirds during northward spring migration, Canada, 2017.

Tick No.	Geographic Location	Prov.	Site	Host	GenBank Accession No.	Lab
17-5A4	Ruthven Park	ON	7	Song Sparrow	MG952944	JEF
17-5A6	Ruthven Park	ON	7	Brown Thrasher	MG958137	JEF
17-5A7B	Ste-Anne-de-Belleville	QC	4	Northern House Wren	MG958138	JEF
CN17-5A32	Toronto	ON	6	Northern House Wren	MH290726	KLC
CN17-5A35	Toronto	ON	6	Ovenbird	MH290727	KLC
CN17-5A38	Ste-Anne-de-Bellevue	QC	4	Northern Waterthrush	MH290728	KLC
CN17-5A39-2	Ste-Anne-de-Bellevue	QC	4	Common Yellowthroat ^†^	MH290729	KLC
CN17-5A42-1	Ste-Anne-de-Bellevue	QC	4	Common Yellowthroat ^†^	MH290730	KLC
CN17-5A51A, C	Swan River	MB	10	Swainson’s Thrush	MH290731–MH290732	KLC
CN17-5A62-2	Long Point	ON	8	Indigo Bunting	MH290733	KLC
CN17-5A74-2	McKellar Island	ON	9	Dark-eyed Junco	MH290734	KLC
CN17-5A77B-2, 3	Toronto	ON	5	Swainson’s Thrush	MH290735–MH290736	KLC
17-5A80A, B	Peace River	AB	12	Swainson’s Thrush	MG967647–MG967648	JEF
17-5A81A, B	Middle River	NS	3	Common Yellowthroat	MG967649–MG967650	JEF
17-5A82B, C	Grand Bank	NL	1	Common Yellowthroat	MG967651–MG967652	JEF
CN17-5A83B	St. Albans	NL	2	Gray-cheeked Thrush	MH 290737	KLC

^†^ photos, see Figure 2.
